# *Pseudomonas aeruginosa* adapts its iron uptake strategies in function of the type of infections

**DOI:** 10.3389/fcimb.2013.00075

**Published:** 2013-11-14

**Authors:** Pierre Cornelis, Jozef Dingemans

**Affiliations:** ^1^Research Group Microbiology, Department of Bioengineering Sciences, Vrije Universiteit BrusselBrussels, Belgium; ^2^Department Structural Biology, VIB, Vrije Universiteit BrusselBrussels, Belgium

**Keywords:** *Pseudomonas aeruginosa*, iron, siderophores, pyoverdine, pyochelin, heme uptake, Feo, phenazines

## Abstract

*Pseudomonas aeruginosa* is a Gram-negative γ-Proteobacterium which is known for its capacity to colonize various niches, including some invertebrate and vertebrate hosts, making it one of the most frequent bacteria causing opportunistic infections. *P. aeruginosa* is able to cause acute as well as chronic infections and it uses different colonization and virulence factors to do so. Infections range from septicemia, urinary infections, burn wound colonization, and chronic colonization of the lungs of cystic fibrosis patients. Like the vast majority of organisms, *P. aeruginosa* needs iron to sustain growth. *P. aeruginosa* utilizes different strategies to take up iron, depending on the type of infection it causes. Two siderophores are produced by this bacterium, pyoverdine and pyochelin, characterized by high and low affinities for iron respectively. *P. aeruginosa* is also able to utilize different siderophores from other microorganisms (siderophore piracy). It can also take up heme from hemoproteins via two different systems. Under microaerobic or anaerobic conditions, *P. aeruginosa* is also able to take up ferrous iron via its Feo system using redox-cycling phenazines. Depending on the type of infection, *P. aeruginosa* can therefore adapt by switching from one iron uptake system to another as we will describe in this short review.

## Pseudomonas aeruginosa

*Pseudomonas aeruginosa* is a ubiquitous γ-proteobacterium found in many diverse environments, such as water or the rhizosphere and it produces a large array of colonization and virulence factors allowing it to establish itself in plants, nematodes, insects, and in mammals, including humans where it can cause different types of infections (Rahme et al., 1997; Mahajan-Miklos et al., 1999; Goldberg, 2000; Lyczak et al., 2000, 2002; Pukatzki et al., 2002). *P. aeruginosa* produces different virulence factors, including the extracellular exotoxin A, extracellular proteases, lipase, phospholipases, and toxins injected via the type III secretion system (Coggan and Wolfgang, 2012; Jimenez et al., 2012; Balasubramanian et al., 2013). The production of many virulence factors is coordinately regulated by small diffusing molecules, via a mechanism termed quorum sensing (Juhas et al., 2005; Schuster and Greenberg, 2006; Venturi, 2006; Williams et al., 2007; Girard and Bloemberg, 2008; Winstanley and Fothergill, 2009). The other important factor allowing colonization of the host is the efficient uptake of iron by the bacterium. In the mammalian host iron is not freely available since it is either present in the heme molecule found in hemoproteins (hemoglobin, cytochromes…) or strongly chelated by extracellular proteins (transferrin and lactoferrin) (Cornelissen and Sparling, 1994). *P. aeruginosa* is able to switch its lifestyle from planktonic unicellular to a sessile form in biofilms (Goodman et al., 2004; Mikkelsen et al., 2011). Two conflicting sensor systems control the switch from planktonic to sessile lifestyle: RetS for the switch to acute virulence with the production of toxins and expression of type III secretion system, and LadS/GacS for the conversion to the sessile mode with the expression of genes involved in type VI secretion system, production of exopolysaccharides, resulting in biofilm formation and chronic infections (Goodman et al., 2004; Mikkelsen et al., 2011; Coggan and Wolfgang, 2012; Balasubramanian et al., 2013). It is not the purpose of this opinion article to go into the details of the mechanisms at the basis of the switch, but rather to emphasize the importance of this duality in relation to iron uptake systems.

## The two oxidation states of iron and their importance for biological systems

Iron is the fourth most abundant element on earth and it is widely used by organisms because it can exist in two oxidation states, Fe^2+^ and Fe^3+^, which is why it is involved in numerous oxido-reduction reactions (Andrews et al., 2003). Fe^3+^ dominates in oxygenated environments, which presents a problem for microorganisms with an aerobic lifestyle because of the extremely low solubility of this form of the metal (Andrews et al., 2003). Conversely, the soluble Fe^2+^ is the most abundant form in anaerobic environments or in microaerobic conditions at low pH (Andrews et al., 2003).

## *Pseudomonas aeruginosa* uses multiple iron uptake systems

As already mentioned, bacterial pathogens are confronted with a problem of iron availability in the host since it is sequestered in the heme molecule or by circulating proteins such as transferrin or lactoferrin (Finkelstein et al., 1983; Cornelissen and Sparling, 1994). Although some pathogens, like *Neisseria*, are able to take-up iron directly from transferrin, this is not an option for *P. aeruginosa* (Cornelissen, 2003; Noinaj et al., 2012).

*P. aeruginosa* can use different strategies to acquire iron:

– Via the production of extracellular Fe^3+^ chelating molecules termed siderophores (pyoverdine and pyochelin) and the uptake of ferrisiderophores via TonB-dependent receptors (TBDR).
– Via the uptake of xenosiderophores (not produced by the bacterium itself).
– Via the uptake of the heme molecule from the host hemoproteins.
– Via the extracellular reduction of Fe^3+^ to Fe^2+^ involving phenazine compounds and a Fe^2+^ dedicated iron uptake system, termed the Feo system.

Depending on the type of infection it causes (acute vs. chronic), *P. aeruginosa* can adapt its iron uptake strategy to best fulfill its needs for the metal without spending too much energy.

## The *P. aeruginosa* pyoverdine and pyochelin-mediated Fe^3+^ uptake systems

Siderophores are low-molecular weight excreted molecules that specifically chelate Fe^3+^ with a high affinity and are taken up by specific receptors dependent on the energy provided by the TonB cytoplasmic membrane protein (Braun and Killmann, 1999; Boukhalfa and Crumbliss, 2002; Hider and Kong, 2011; Schalk et al., 2012; Schalk and Guillon, 2013). Siderophores are of different types, based on the way the iron is complexed: phenolate-, catecholate-, hydroxamate-, carboxylate-, or mixed type of siderophores have been described.

### Pyoverdines: the high affinity siderophores are needed to cause acute infections

**Figure 1:**
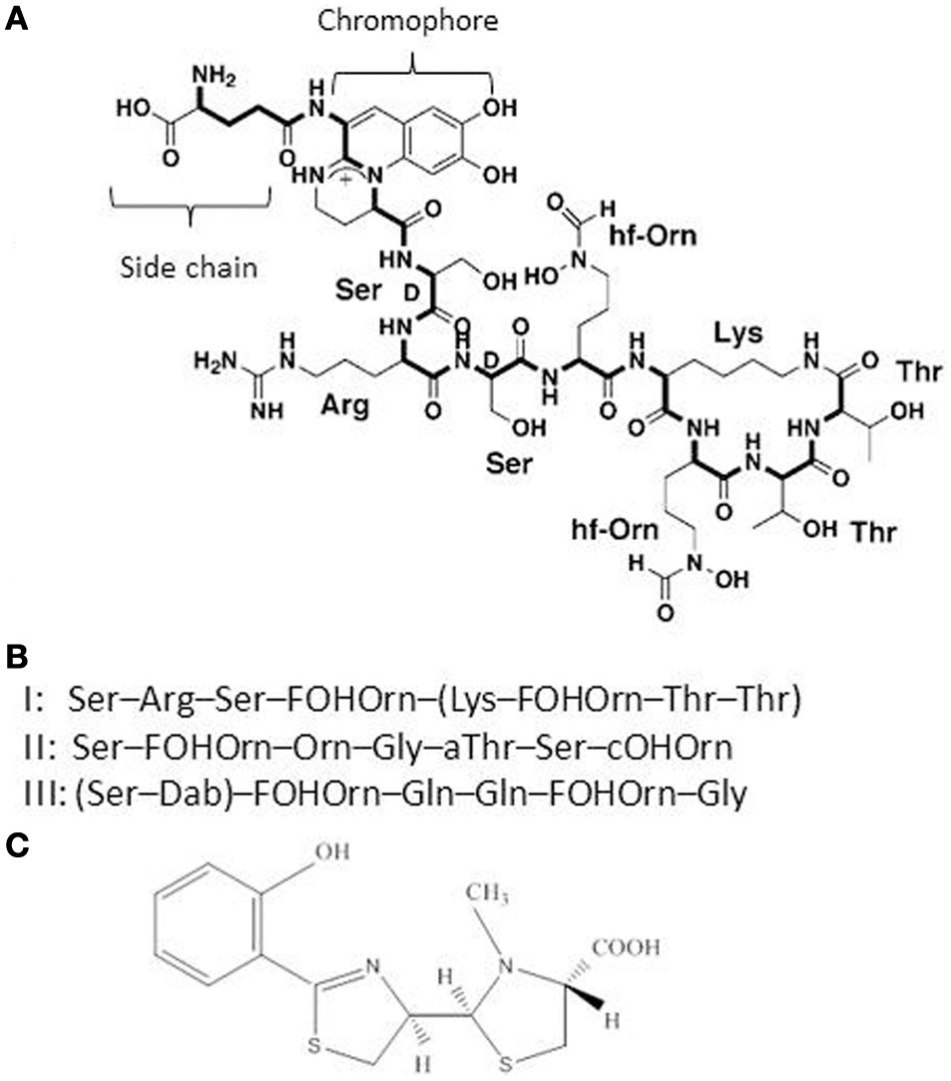
**(A)** Structure of the *P. aeruginosa* type I pyoverdine [taken from Ravel and Cornelis (2003)]. **(B)** Sequence of the peptide chains from *P. aeruginosa* type I, II, and III pyoverdines. **(C)** Structure of the second *P. aeruginosa* siderophore pyochelin.

**Figure 2:**
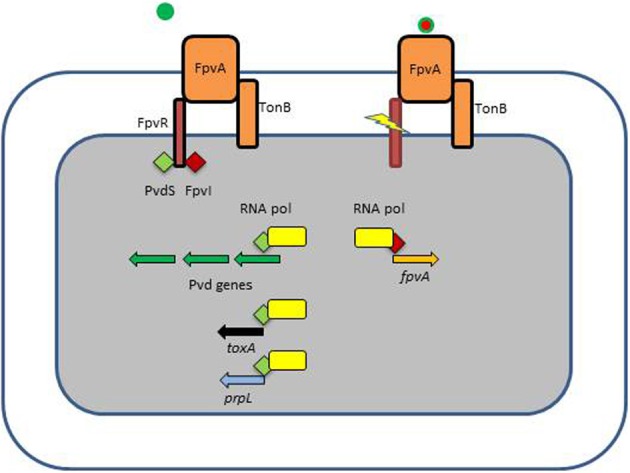
**Ferripyoverdine is also a signal molecule**. The left panel shows the FpvA receptor empty since only apo-pyoverdine is present. FpvA is a TonB-dependent receptor which is also associated with the FpvR anti-σ factor. FpvR sequesters two ECF σ, PvdS and FpvI via its cytoplasmic domain. In this condition there is little possibility for the respective σ factors to associate with the core RNA polymerase and the pyoverdine biosynthesis genes are not transcribed (PvdS) neither the *fpvA* gene (FpvI). The right panel shows what happens when ferripyoverdine binds to the FpvA receptor. This binding triggers a conformational change resulting in the proteolysis of FpvR, which liberates the two σ factors which now can associate with the RNA polymerase. PvdS is not only involved in the transcription of pyoverdine genes, but also in the expression of two virulence genes, *prpL* encoding an extracellular protease and *exoA* encoding the potent exotoxin A.

*P. aeruginosa* pyoverdine is a composite (mixed) siderophore comprising a peptide chain and a chromophore (Meyer, 2000; Ravel and Cornelis, 2003; Visca et al., 2007) and the structure of one *P. aeruginosa* pyoverdine (type I) is shown in Figure 1. Pyoverdines are the hallmark of fluorescent *Pseudomonas* species (*P. fluorescens*, *P. putida*, *P. syringae*, *P. aeruginosa*) and are produced when the bacteria are grown in low iron conditions (Meyer, 2000; Ravel and Cornelis, 2003; Visca et al., 2007). The conserved chromophore part of the molecule provides the catecholate function participating in the binding of Fe^3+^ while the peptide chain is highly variable among representatives of the different *Pseudomonas* species or even within a species (Meyer et al., 2008). Pyoverdines peptide chains contain between 6 and 12 amino-acids (Meyer, 2000; Ravel and Cornelis, 2003; Visca et al., 2007). Within the species *P. aeruginosa*, three different types of pyoverdines have been recognized, which differ by the composition of their respective peptide chain (Cornelis et al., 1989; Meyer et al., 1997). Pyoverdines bind iron with a very high affinity, are able to displace iron from transferrin and pyoverdine production is absolutely needed to cause infection in a burned mouse model or in case of mouse pulmonary infections (Albrecht-Gary et al., 1994; Meyer et al., 1996; Meyer, 2000; Takase et al., 2000a,b; Imperi et al., 2013). Likewise, a mutant in the TonB protein is avirulent in a mouse model (Takase et al., 2000b). In their recent study, Imperi et al. screened different FDA-approved compounds for their inhibitory activity toward pyoverdine production by *P. aeruginosa*. One such compound, the antimycotic drug flucytosine, caused a strong decrease in pyoverdine production in several strains of *P. aeruginosa* producing the different pyoverdine types and the inhibitory activity was found to target the extracytoplasmic sigma factor (ECF σ) PvdS, which is needed for the transcription of pyoverdine biosynthesis genes (Imperi et al., 2013). Pyoverdine is not only a siderophore, but also a signal molecule since it triggers the production of two extracellular virulence factors, the protease PrpL and the potent toxin exotoxin A (Figure 2) (Lamont et al., 2002; Redly and Poole, 2003, 2005; Visca et al., 2007; Cornelis, 2010). Although pyoverdine seems to be essential to *P. aeruginosa* to cause acute infections, it has also been shown to be involved in the establishment of thick mature biofilms (Banin et al., 2005; Patriquin et al., 2008; Glick et al., 2010).

### Pyochelin: the low-affinity siderophore

Pyochelin (Figure 1), the second siderophore of *P. aeruginosa*, is produced by all *P. aeruginosa* isolates, but its affinity for iron is much lower compared to pyoverdine (Cox et al., 1981; Ankenbauer et al., 1988; Brandel et al., 2012). Pyochelin biosynthesis involves a lower number of genes compared to pyoverdine (Serino et al., 1997) and it has been recently demonstrated that *P. aeruginosa* first produces pyochelin and switches to pyoverdine production only when the concentration of iron becomes really low (Dumas et al., 2013). Pyochelin-iron can redox-cycle and has been shown to cause oxidative damage and inflammation, especially in the presence of another *P. aeruginosa* extracellular compound, pyocyanin (Coffman et al., 1990; Britigan et al., 1992, 1997). In chronic infections, such as in CF lungs, the production of pyochelin could play a role in the sustained inflammatory response which is known to occur and cause damage to tissues (Lyczak et al., 2002). It has been shown that pyochelin production is increased in a synthetic CF sputum medium (Hare et al., 2012).

## Heme uptake systems

**Figure 3:**
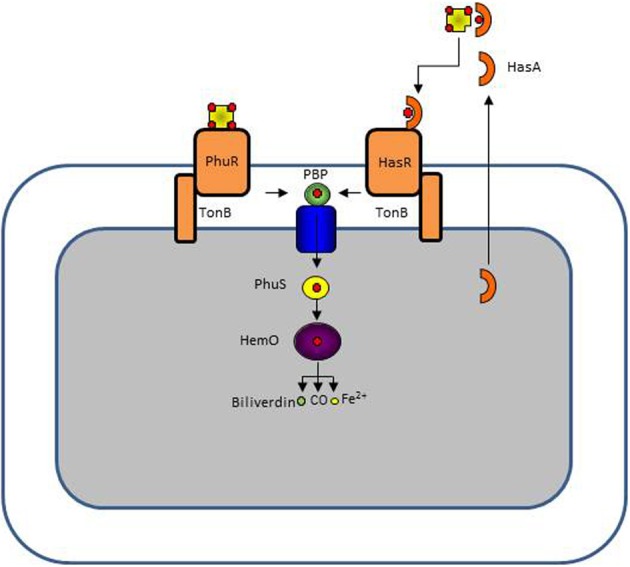
***P. aeruginosa* has two heme uptake systems, Phu, and Has**. The PhuR TonB-dependent receptor binds directly hemoproteins extracting heme while the HasR receptor binds heme complexed to a secreted hemophore protein HasA. In the periplasm heme is bound to a periplasmic protein which delivers it to an ABC transporter. In the cytoplasm heme is directed to the heme oxygenase HemO by the PhuS chaperone. HemO cleaves the tetrapyrrole ring, leaving biliverdin, CO, and Fe^2+^.

*P. aeruginosa* has the capacity to take up heme from hemoproteins via the two systems Has and Phu (Ochsner et al., 2000). Heme is not found in its free form because it is highly hydrophobic causing it to associate with membranes where it promotes non-enzymatic redox reactions (Wyckoff et al., 2005). Heme must therefore be extracted from hemoproteins such as hemoglobin or hemopexin. In the *P. aeruginosa* Phu system heme is directly extracted by an outer membrane TBDR while in the Has system, heme is first extracted by a secreted protein, the hemophore. The hemophore-heme complex is recognized by another TBDR, HasR (Letoffe et al., 1998; Wandersman and Delepelaire, 2004, 2012). Once in the periplasm, heme is bound by a periplasmic binding protein and transported to the cytoplasm by an ABC transporter where it is first bound to a heme chaperone, PhuS, before being delivered to the heme oxygenase HemO where the heme molecule will be degraded to form biliverdin, CO, and Fe^2+^ (Bhakta and Wilks, 2006; Lansky et al., 2006; Barker et al., 2012; O'Neill et al., 2012). A single mutant in the *phu* system or in the *has* system is still able to take up heme while a double *phu has* mutant is virtually unable to use heme as a source of iron (Ochsner et al., 2000). The Phu and Has systems are schematically presented in Figure 3.

## Uptake of xenosiderophores

*P. aeruginosa* strains have generally more than 30 genes encoding TBDRs, the majority of them involved in the uptake of ferrisiderophores (Bodilis et al., 2009; Cornelis and Bodilis, 2009; Cornelis et al., 2009). The different TBDRs can be classified into two categories, the simple TBDR and the TonB-dependent transducers (TBDT) (Hartney et al., 2011). The TBDT, of which the ferripyoverdine receptor FpvA is an example, can sense the presence of the cognate ferrisiderophore by interacting with a membrane protein which acts as an anti-sigma factor (Hartney et al., 2011). Upon recognition of the ferrisiderophore by the cognate receptor, the anti-sigma factor undergoes a proteolytic degradation liberating the extracytoplasmic sigma factor (ECF σ), which associates with the RNA polymerase to transcribe the receptor gene, causing an auto-induction reaction (Cornelis et al., 2009; Mettrick and Lamont, 2009; Cornelis, 2010; Hartney et al., 2011). The majority of *P. aeruginosa* strains (98%) have a second receptor for type I ferripyoverdine, FpvB, which means that almost all strains have the capacity to use this pyoverdine as source of iron (Ghysels et al., 2004; Bodilis et al., 2009). *P. aeruginosa* is also able to utilize the *E. coli* siderophore enterobactin via two different receptors, PfeA and PirA since only a double *pfeA pirA* mutant is unable to take up ferrienterobactin (Dean and Poole, 1993; Ghysels et al., 2005; Cornelis and Bodilis, 2009). Other characterized receptors are FoxB and FiuA for the uptake of ferrioxamine and ferrichrome (Llamas et al., 2006; Cuiv et al., 2007; Banin et al., 2008; Hannauer et al., 2010), FemA for the utilization of mycobactin and carboxymycobactin (Llamas et al., 2008), FecA for Fe-citrate uptake (Marshall et al., 2009), ChtA for rhizobactin, aerobactin, and schizokinen (Cuiv et al., 2006), and FvbA for the uptake of vibriobactin (Elias et al., 2011). However, the importance of these xenosiderophore uptake systems in infections has not, to the best of our knowledge, been established. They could however be of importance in case of polymicrobial infections where *P. aeruginosa* could be at advantage because of its capacity to steal siderophores produced by other microorganisms (siderophore piracy) (Traxler et al., 2012) while depriving the competitors from iron because they would be unable to recognize the complex pyoverdine siderophore.

## Uptake of Fe^2+^ via the feo system: involvement of phenazines

**Figure 4:**
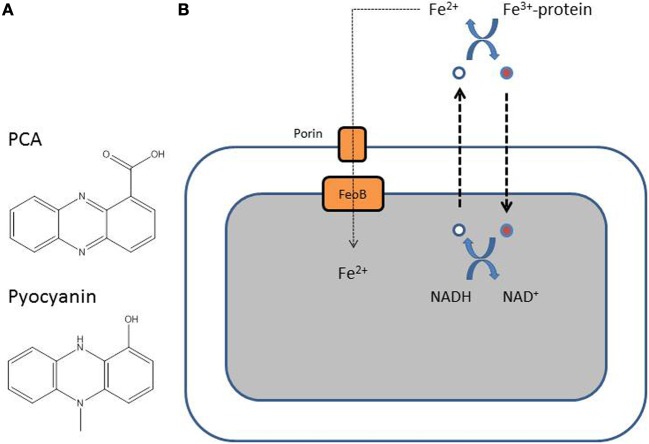
**(A)** Structure of the two major *P. aeruginosa* phenazines, phenazine-1-carboxylic acid (PCA), and pyocyanin. **(B)** Reduced PCA (white-filled circle) is excreted out of the cell and is oxidized (red-filled circle) resulting in the reduction of Fe^3+^ to Fe^2+^. The oxidized phenazine is recycled inside the cell where it is reduced again with simultaneous oxidation of NADH to NAD (Wang et al., 2011).

Unlike Fe^3+^, Fe^2+^ is soluble and is present in anaerobic conditions or in microaerobic environments at lower pH (Andrews et al., 2003). Fe^2+^ probably diffuses through the outer membrane and is further transported inside the cytoplasm by the FeOABC system, which is present in many Gram-negative bacteria (Cartron et al., 2006). The soluble Fe^2+^ is transported inside the cells via a transport system composed of the permease FeoB, and the proteins FeoA and FeoC (Cartron et al., 2006). The uptake of Fe^2+^ by *P. aeruginosa* is probably relevant when the bacterium finds itself in a microaerobic or anaerobic environment, a situation known to exist in the CF lung mucus where *P. aeruginosa* forms biofilms (Worlitzsch et al., 2002; Yoon et al., 2002). Phenazines are secondary metabolites produced by *P. aeruginosa* (Figure 4). Phenazine-1-carboxylic acid (PCA) is the precursor of pyocyanin, a blue-green compound typical of *P. aeruginosa*, and both phenazine compounds can redox-cycle (Wang and Newman, 2008). PCA, and to a lesser extent pyocyanin, is able to reduce Fe^3+^ bound to host proteins to Fe^2+^, allowing the uptake of iron in biofilms via the Feo system (Figure 4) (Wang et al., 2011). Recently, it was demonstrated that both phenazines and Fe^2+^ accumulate in the lungs of CF patients when their condition deteriorates (Hunter et al., 2012, 2013). In their last article, Hunter et al. (2013) also show that maximal *P. aeruginosa* biofilm disruption is achieved using a combination of both Fe^3+^ and Fe^2+^ chelators.

## Adaptation of *P. aeruginosa* iron uptake strategies: the example of CF lung infections

As already mentioned, *P. aeruginosa* can switch from the production of pyochelin, the low affinity siderophore, to the more energy demanding high affinity pyoverdine in function of the availability of Fe^3+^ (Dumas et al., 2013). One typical example is the adaptation of *P. aeruginosa* to the CF lung environment (Lyczak et al., 2002). When *P. aeruginosa* invades the lungs, it is probably able to produce pyoverdine, but with longer colonization times the bacterium induces a strong inflammatory response, due to the production of pyochelin among other causes, resulting in tissue damage and release of cellular contents, including hemoproteins and other iron-containing proteins (Britigan et al., 1997). Although siderophores, including pyoverdine, have been detected in the sputum samples of CF patients (Martin et al., 2011), pyoverdine-negative mutants accumulate with longer times of colonization (De Vos et al., 2001; Lamont et al., 2009), suggesting that alternative systems are used by *P. aeruginosa* to fulfill its needs for iron. This is the case since *P. aeruginosa* can take up heme from hemoproteins released by the inflammatory process and Fe^2+^ generated by the redox activity of phenazines, in particular PCA (Lamont et al., 2009; Wang et al., 2011; Hunter et al., 2013; Konings et al., 2013).

## Conclusions

From the analysis of the abundant literature on *P. aeruginosa* iron uptake systems, it is clear that this bacterium can exquisitely adapt its iron capture strategy in function of the type of infection it causes. When causing acute infections, it uses its high-affinity pyoverdine siderophore, which at the same time acts as a signal molecule for the production of acute virulence factors. On the other hand, when establishing itself in a niche where it can persist and cause inflammation, it tends to lose its capacity to produce pyoverdine and to rely on alternative iron uptake strategies, including the uptake of heme from hemoproteins and the uptake of Fe^2+^ generated via the redox activity of phenazines. This has certainly implications in terms of finding treatments based on iron chelation since not only Fe^3+^ scavenging should be considered (Ballouche et al., 2009; Hunter et al., 2013).

### Conflict of interest statement

The authors declare that the research was conducted in the absence of any commercial or financial relationships that could be construed as a potential conflict of interest.
